# The interplay of configuration and conformation in helical perylenequinones: Insights from chirality induction in liquid crystals and calculations

**DOI:** 10.3762/bjoc.8.16

**Published:** 2012-01-24

**Authors:** Elisa Frezza, Silvia Pieraccini, Stefania Mazzini, Alberta Ferrarini, Gian Piero Spada

**Affiliations:** 1 Department of Chemical Sciences, University of Padova, via Marzolo 1, 35131 Padova, Italy; 2 Dipartimento di Chimica Organica “A. Mangini”, Alma Mater Studiorum – Università di Bologna, via San Giacomo 11, 40126 Bologna, Italy; 3 Dipartimento di Scienze Molecolari Agroalimentari, Università degli Studi di Milano, via Celoria 2, 20133 Milano, Italy

**Keywords:** chirality, conformational analysis, DFT calculations, helical twisting power, nematic liquid crystals

## Abstract

The chirality transfer in liquid crystals induced by two helical perylenequinones (namely, the natural compounds cercosporin and phleichrome) was investigated by integrating measurements of helical twisting power with a conformational analysis by DFT calculations and with the prediction of their twisting ability by the surface-chirality method. The two *quasi*-enantiomeric derivatives induce oppositely handed cholesteric phases when introduced as dopants in nematic solvents. We evaluated the role of the different conformations of the chiral hydroxyalkyl side chains in determining the helical twisting power: They were found to affect the strength of the chirality transfer, although the handedness of the induced cholesteric phase is essentially determined by the axial chirality (helicity) of the core of the perylenequinones.

## Introduction

The phenomenon of chiral induction in nematic mesophases has been known for a long time [1]. By addition of a chiral nonracemic compound, a nematic liquid crystal is transformed into a chiral nematic (or cholesteric) phase. Here the director, i.e., the local alignment direction, rotates in space in helical way, along a perpendicular axis [2–3]. The handedness of this helix reflects the configuration of the dopant: Enantiomers induce oppositely handed cholesterics. Only in the last few decades has the generation of cholesteric liquid crystals and the amplification of the molecular chirality observed upon doping nematic phases with chiral derivatives attracted great interest in the field of material science [3–4]. In this context, one of the major research lines focuses on the investigation of the chirality transfer between “shape persistent” dopants and nematic solvents [2,5–11]. Thus, the chirality amplification from the molecular to mesophase level can be exploited for the determination of the absolute configuration [2–3,5–6,12–24]. In fact, this technique has been fruitfully applied to different classes of systems, possessing either stereogenic centers or axial chirality.

**Figure 1:**
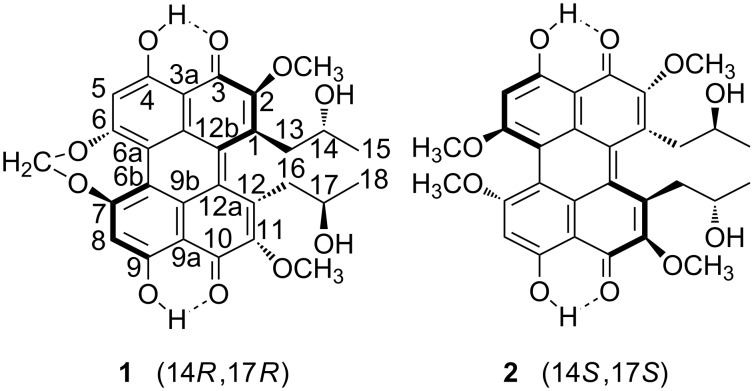
Chemical structure of the helical perylenequinones under investigation: Cercosporin (**1**) and phleichrome (**2**).

In this work, chirality induction in liquid crystals has been used for a structural study of helical perylenequinones. This is an important family of natural products, characterized by the presence of a helical chiral conjugated pentacyclic core [25]. These systems have attracted considerable attention due to their photosensitizing properties and their phytotoxic activity. Another reason for the interest in perylenequinones is their peculiar structural properties, which require special strategies for the complete structural determination. In particular we have focused on the two helical perylenequinones, cercosporin (**1**), [26] and phleichrome (**2**) [27], shown in Figure 1. They have the same stereochemical features: Two bulky methoxy groups or a strained seven-membered ring in positions 6 and 7, two side chains in positions 1 and 12 and a nonplanar helical shape. The helicity generates axial chirality, which, when associated with the presence of asymmetrically substituted carbon atoms in the side chains, gives rise to diastereoisomerism. Cercosporin and phleichrome are characterized by a special coupling between conformation and configuration: The conformational preferences of the side chains in the “1–12 bay region” are critical for the generation of the helical structure. X-ray crystallography established the *R*,*R* configuration at C14 and C17 of cercosporin (**1**) and the sign of the axial chirality as *M* [26,28]. Phleichrome (**2**) features opposite chirality, having *P* axial chirality and *S*,*S* configuration at C14 and C17 [27].

The ring substituents give **1** and **2** a limited, though non-negligible conformational freedom. To understand the relation between molecular structure and chiral induction in liquid crystals, we integrated measurements of helical twisting power (HTP) with a conformational analysis, performed by density functional theory (DFT) calculations, and with the prediction of the twisting ability of conformers, by the surface chirality (SC) method [29].

## Results and Discussion

### HTP measurement

The propensity of a dopant to induce a helical organization in the liquid-crystalline matrix is measured by its helical twisting power, which is defined as


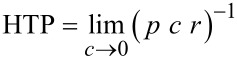


where *p* is the helical pitch (in μm) of the cholesteric phase, and *c* and *r* are the concentration (molar fraction) and the enantiomeric excess of the dopant, respectively. The sign of HTP is taken as positive or negative if the induced cholesteric is right- or left-handed, respectively. The HTP of **1** and **2** was measured in the liquid-crystal mixture E7 [30] at a temperature of 298 K. The values −12 μm^−1^ and +54 μm^−1^ were obtained for **1** and **2**, respectively. The opposite sign observed for the two compounds clearly reflects their opposite configuration. The handedness of the induced cholesterics is that which is expected for helicoidal disc-like dopants, as binaphthyl derivatives [20] and helicene-like molecules [22,24], i.e., left-handed for the *M* and right-handed for the *P* molecular helicity. In view of the similar molecular shape of **1** and **2**, the difference between the absolute values of their HTP is somewhat surprising. As a possible explanation for this difference we can devise a different conformation of the aromatic core in the two compounds or a different arrangement of the substituents, in particular of the chiral hydroxyalkyl side chains (henceforth “the side chains”). To explore this issue we performed a computational study at different levels: Single-molecule DFT calculations were carried out to evaluate energy and geometry of all the conformers of **1** and **2**, and the SC method was used to estimate their twisting ability.

### Conformational analysis by DFT

**Figure 2:**
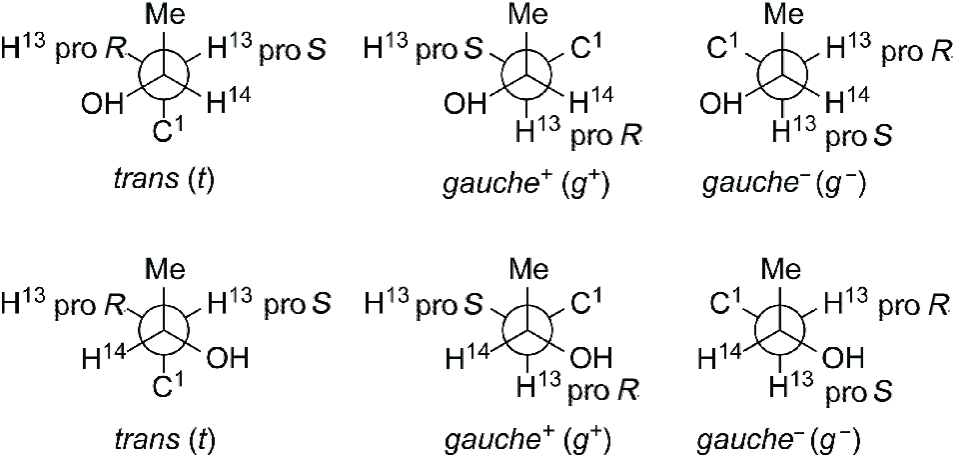
Newman projections of the conformational states of the side chain linked at C1 of cercosporin (**1**), with 14*R* configuration (top), and of phleichrome (**2**)**,** with 14*S* configuration (bottom). Analogous states exist for the other chain, linked at C12.

For each of the side chains in position 1 and 12 of cercosporin and phleichrome, three conformational states are possible, which are shown in Figure 2, where the same notation as in [31] is used. This makes a total of six conformers for each molecule, which are labeled according to the state of each side chain; thus, for instance, *g^+ ^t* is a conformer with one chain in the *gauche*^+^ and the other in the *trans* state. The conformers with side chains in different states are two-fold degenerate: *g^+ ^t* (*= t g^+^*), *g^+ ^g^−^* (= *g^− ^g^+^*) and *g^− ^t* (*= t g^−^*)*.*

For the conformational study, we used DFT calculations in vacuum as implemented in the Gaussian suite of programs [32–33]. We selected the hybrid functional B3LYP [34] with the 6-31g(d,p) basis set, which is a standard choice and is relatively inexpensive from the computational point of view. Then, considering that dispersive interactions between the side chains and the aromatic ring could be crucial for the systems under investigation**,** further calculations were carried out with the functional M06-2X [35], which was developed recently to provide a better description of nonlocal electronic correlation with respect to standard functionals. In this case the more demanding 6-31g+(d,p) basis set was used. Geometry optimization of all the conformers of **1** and **2** was carried out. In the starting configurations, the methoxy substituents were taken always in the same orientation, which was found to be only slightly modified in the optimized geometry. The C–O–C bonds of the methoxy groups in the “6,7-bay region” of **2** lie in the plane of the adjacent aromatic ring, in agreement with the torsional potential of anisole [36]. For steric reasons, a planar arrangement is not possible for the methoxy substituents at the 2 and 11 positions. In general, two orientations are allowed for each methoxy group, with torsional angles in the ranges +(110°–145°) (*p* states) and −(110°–145°) (*m* states). Thus, we can distinguish four different states of the methoxy groups, labelled as (*m*,*m*), (*m*,*p*), (*p*,*m*) and (*p*,*p*). To limit the computational cost, we only considered the (*p*,*p*) states. This is the state found in one of the available X-ray structures of cercosporin [26], whereas the other structure has the methoxy groups in the (*m*,*p*) state [28].

**Figure 3:**
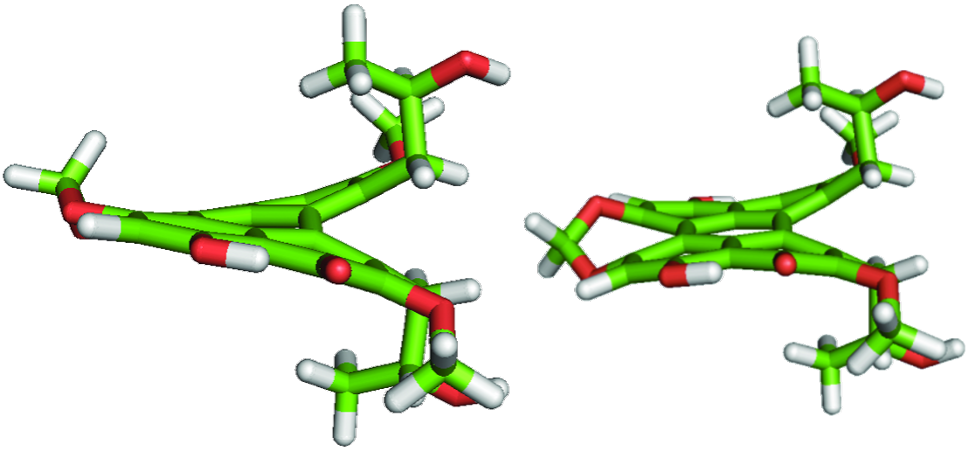
“Propeller” (left) and “double butterfly” geometry (right) of the *g*^+^* g*^+^ conformer of **1**, as obtained by geometry optimization by DFT at the M06-2X/6-31+g(d,p) level [33].

Our calculations confirm that the “propeller” form, found in X-ray structures of **1** [26,28], is significantly more stable than the other, called “double butterfly” by Falk and co-workers [37]. The two geometries, as obtained for the *g*^+^ *g*^+^ conformer of **1,** are shown in Figure 3. Table 1 reports the twist angles χ_1_ [C(1)–C(12b)–C(12a)–C(12)] and χ_2_ [C(6)–C(6a)–C(6b)–C(7)], which characterize the helical shape of the core of **1** and **2**. Not surprisingly, in view of the opposite configuration, the twist angles of the propeller form of **1** and **2** have opposite signs. We have found that χ_1_ and χ_2_ have a weak dependence on the side chain conformation. Our results are in good agreement with X-ray data for cercosporin [26,28], whereas for phleichrome no structural data are available. However, the prediction that χ_1_ ~ χ_2_ ~ 30° appears reasonable for **2**, considering that the narrower χ_1_ angle in cercosporin is a consequence of the constraints imposed by the bridge in the “6,7-bay region”.

**Table 1:** Twist angles for **1** and **2**, as obtained by DFT geometry optimization and by crystallography. The “propeller” form is considered, unless otherwise specified (the symbol *db* denotes the “double butterfly” geometry).

compound	method	χ_1_ (°)	χ_2_ (°)

**1**^a^	DFT-B3LYP/6-31g(d,p)	−30.0 to −30.9	−11.8 to −12.6
	DFT/M06-2X/6-31+g(d,p)	−29.0 to −29.7	−13.1 to −12.0
**1** (*db*)^b^	DFT/M06-2X/6-31+g(d,p)	−26.4	18.1
**1**^a^	X-ray [28]	−29.8	−9.2
	X-ray [26]	−27.4	−9.9
**2**^b^	DFT-B3LYP/6-31g(d,p)	32.4 to 34.0	30.8 to 31.6
	DFT/M06-2X/6-31+g(d,p)	32.0 to 33.1	29.6 to 30.3

^a^range of angles for six side-chain conformers; ^b^*g*^+^*g*^+^ conformer.

**Figure 4:**
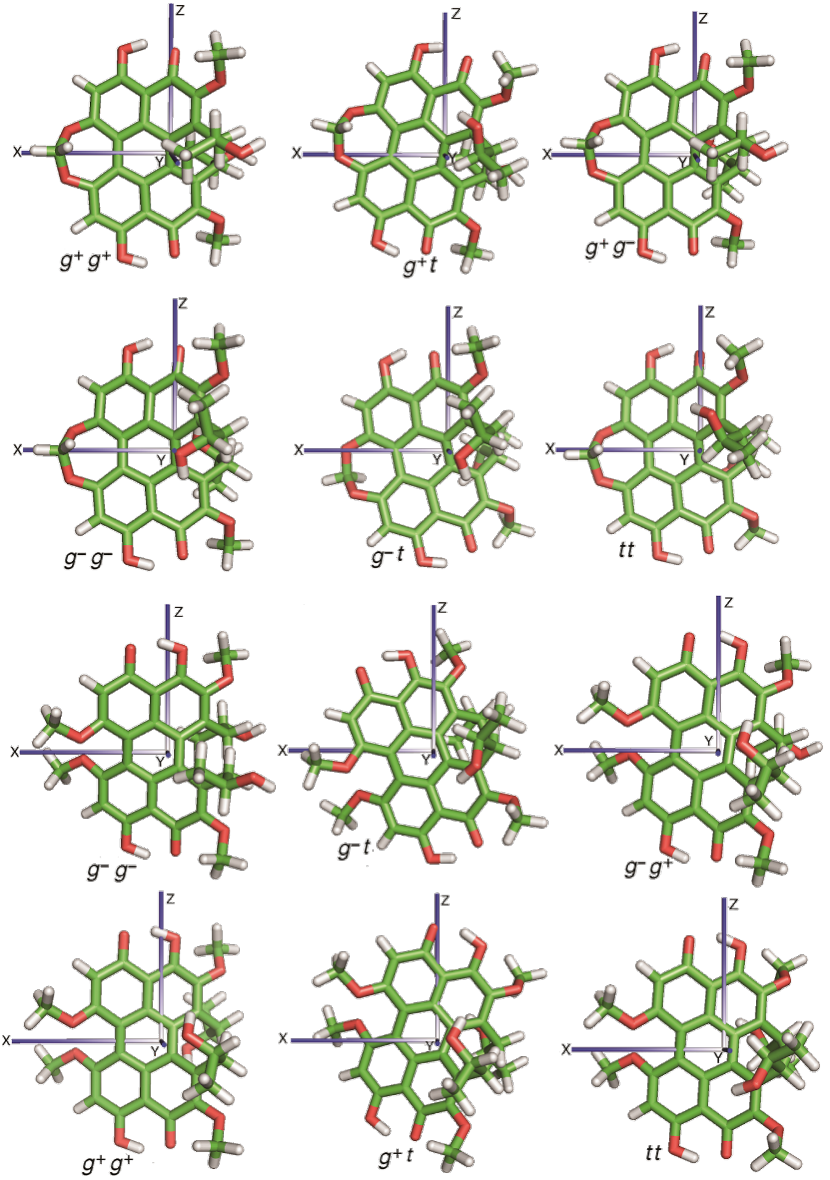
Optimized geometry of all conformers of **1** (upper half) and **2** (lower half), obtained at the DFT/M06-2X/6-31+g(d,p) level [33]. Superimposed on each structure, the principal axes (*x*,*y*,*z*) of the Saupe matrix calculated by the SC method [29] are shown. The reference frame is defined in such a way that the *z* and *y* axes have the highest tendency to lie parallel and perpendicular to the director, respectively.

In view of their higher stability, only conformers in the propeller form were considered in our systematic analysis of the effects of side-chain conformations. Very similar structures were obtained by geometry optimization at the B3LYP/6-31g(d,p) and at the M06-2X/6-31+g(d,p) level; the latter are shown in Figure 4. On the contrary, the conformer energies were found to depend strongly on the level of calculation, as shown in Table 2. Significant differences between conformers were predicted at the B3LYP/6-31g(d,p) level: The *t t* was strongly preferred and either *g*^−^ or *g*^+^ states were found to have a highly destabilizing effect for **1** and **2**, respectively. Much smaller differences in conformer stability were obtained at the M06-2X level. To check whether the discrepancies between the two kinds of calculations were mainly due to the functional or due to the basis set, we also performed a few calculations for **2** at the B3LYP/6-31+g(d,p) level. With the new basis we found a significant decrease of the energy differences between conformers, which points to an important role of diffuse functions.

**Table 2:** Energy of the conformers of **1** and **2**, obtained by geometry optimization by DFT at the (a) B3LYP/6-31g(d,p) and (b) M06-2X/6-31+g(d,p) level of the theory. Within each set, the most stable conformer is taken as the reference (E = 0).

conformer	E **1** (a) [kJ/mol]	E **1** (b) [kJ/mol]	conformer	E **2** (a) [kJ/mol]	E **2** (b) [kJ/mol]

*g*^+ ^*g*^+^	7.7	0.3	*g*^− ^*g*^−^	9.8	0
*g*^+ ^*t*	4.7	0.8	*g*^− ^*t*	5.3	1.4
*t t*	0	0	*t t*	0	0.1
*g*^+ ^*g*^−^	13.3	3.2	*g*^− ^*g*^+^	16.4	3.6
*g*^− ^*t*	9.6	2.5	*g*^+^ *t*	10.8	1.8
*g*^− ^*g*^−^	18.2	3.4	*g*^+^ *g*^+^	22.5	6.8

Although the conformer population is not directly accessible, we can try to analyze our results in the light of experimental data. Of the two X-ray structures of cercosporin reported in the literature, one has the side chains in the *g*^+^* g*^+^ [28] and the other in the *g*^−^ *g*^−^ conformation [26]. These do not appear to be compatible with the strong preference for the *trans* state predicted by B3LYP calculations, but the conformational preferences in crystals might be biased by the environment. More suitable information on the molecular conformation in solution can be obtained from NMR-NOE experiments [31]; however, these do not provide the population of each single conformer but only the overall probability of *t*, *g*^+^ and *g*^−^ states around bonds C(13)–C(14) and C(16)-C(17). For ease of comparison, we have collected these probabilities for compounds **1** and **2** in Table 3, together with estimates based on our DFT calculations. Experimentally, a preference for *gauche*^+^ (for **1**) and *gauche^–^* (for **2**) was inferred. This is in strong contrast with our B3LYP predictions. The M06-2X results are closer to the experimental data, although non-negligible differences appear: For **1** the contribution of *gauche*^+^ states is underestimated, mainly at the expense of the *gauche*^−^ states, and the discrepancies are even larger for **2**, which is predicted to have a prevalence of *trans* states, whereas experimentally a net prevalence of *gauche^−^* states was found. A possible reason for the lack of agreement between theory and experiment is the fact that calculations were performed in vacuum, whereas experiments were carried out in acetone. According to our calculations, the conformers of phleichrome (**2**) would have higher dipole moment than those of cercosporin (**1**), therefore **2** should be more sensitive to solvent polarity (acetone has a dielectric constant of about 20 at room temperature).

**Table 3:** Probability of the three conformational states around bonds C(13)–C(14) and C(16)–C(17) for compounds **1** and **2**, as obtained by our DFT calculations and by NOE experiments [31].

compound	method	probability		
		*trans*	*gauche*^+^	*gauche*^−^

**1**	B3LYP/6-31g(d,p)	0.84	0.14	0.02
**1**	M06-2X/6-31+g(d,p)	0.37	0.40	0.23
**1**	NOE [19]	0.35	0.53	0.15
**2**	B3LYP/6-31g(d,p)	0.87	0.01	0.12
**2**	M06-2X/6-31+g(d,p)	0.42	0.23	0.35
**2**	NOE [31]	0.34	0.13	0.58

### HTP predictions by the SC method

Within the SC approach, the HTP of a chiral dopant in a nematic solvent is proportional to the so-called chirality parameter *Q*, which is defined in terms of the helicity of the molecular surface and the orientational order of the dopant [29]. The proportionality factor between HTP and *Q* depends on the macroscopic properties of the host. Therefore, it is the same for different dopants in the same host; typical values of this factor are about 2–3 [2].

**Figure 5:**
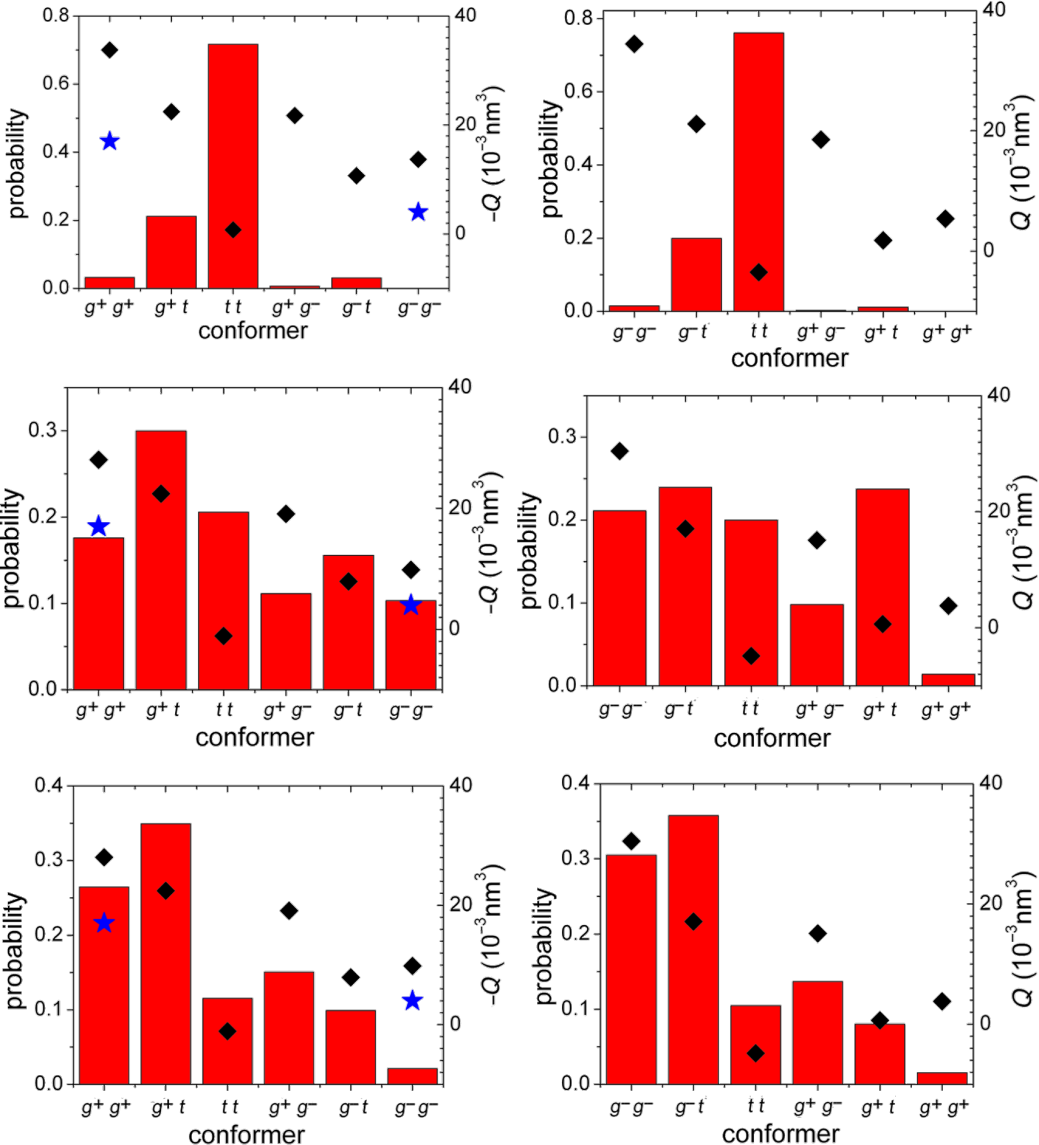
Chirality parameter *Q* (diamonds) and probability distribution (bars), calculated for all conformers of **1**, on the left, and **2**, on the right. Conformer geometry and energy were obtained by DFT calculations at the B3LYP/6-31g(d,p) (top) and the M06-2X/6-31+g(d,p) level (middle). The plots on the bottom report the *Q* values obtained with the geometry calculated at the M06-2X/6-31+g(d,p) level, along with conformer probability distributions inferred from NOE measurements [31]. Stars represent the *Q* values calculated for the X-ray structures of **1** [26,28]. For ease of comparison, the values are reported as the negative of *Q* for **1**.

We calculated the chirality parameter *Q* of the conformers of **1** and **2**, using the molecular geometries obtained by DFT, with either the B3LYP or the M06-2X functional. The results are shown in Figure 5, together with the probability distribution of conformers. We can see in the figure that the side-chain conformation can significantly affect the chirality parameter. For the sake of comparison, we calculated also the chirality parameter of the bare cores, obtaining *Q* = −7·10^−3^ nm^3^ for **1** and *Q* = +9·10^−3^ nm^3^ for **2**. These opposite values are in line with the fact that the two cores are almost the mirror image of each other. In fact, we can see in Figure 5 that the same relation remains for the whole molecules: The *Q* value predicted for a given conformer of **1** is close in magnitude to the *Q* value for the conformer of **2** that is nearly its mirror image, but opposite in sign. The magnitude of *Q* for the cores lies within the values obtained for the various conformers of the whole molecule: Depending on their orientation, side chains were found to either enhance or weaken the twisting ability of the core. The sign of the chirality parameter *Q* can be easily explained on the basis of the chirality and the orientational behavior of the two perylenequinones. All conformers are predicted to preferentially orient with the normal to the aromatic rings lying perpendicular to the nematic director, and with some preference for aligning to the director their z axis, whose direction in the molecule slightly depends on the chain conformation (Figure 4). Thus they convey to the phase the molecular helicity along the molecular *y* axis (perpendicular to the aromatic rings), which is left-handed for cercosporin (**1**) and right-handed for phleichrome (**2**).

It is worth remarking that the results shown in Figure 5 were obtained for the six structures, differing in the conformation of the side chains at positions 1 and 12, and all having the methoxy substituents in the (*p*,*p*) state. Explorative calculations, at the M06-2X/6-31+g(d,p) level, were performed for selected conformers of **2**, with the methoxy substituents in (*m*,*m*), (*m*,*p*) and (*p*,*m*) states. It was found that the state of the methoxy groups can affect the relative energy (up to a couple of kJ/mol) and, to a lesser extent, the *Q* parameter differences between conformers; however, it does not dramatically modify the trend reported in Figure 5 (middle).

We have also shown in Figure 5 (left) the *Q* values calculated for the available X-ray structures of cercosporin [26,28]. The differences from the results reported for structures obtained by DFT, with the same conformation of the side chains, derive from relatively small changes in the molecular geometry.

Table 4 reports the HTP values measured for **1** and **2**, along with the *Q* values, calculated for the two compounds by averaging over all conformers (see Experimental). Negative and positive helical twisting power are predicted for **1** and for **2**, respectively, in agreement with experiments. However, the *Q* parameters do not scale with the measured HTPs: Whereas the absolute value of the HTP of phleichrome is about four times as big as that of cercosporin, the *Q* value calculated for **2** is smaller than that of **1**. We supposed that the main reason for these differences could be the unsatisfactory conformer distributions, which were used to calculate the average chirality parameters. Thus, on moving from the distributions derived from B3LYP to those from M06-2X calculations, the ratio between the absolute values of *Q* for **1** and **2** decreases, and a further decrease can be seen when the NOE probabilities are used. However, this ratio remains far from the experimental value, due to an overestimate of the magnitude of the chirality parameter *Q* for cercosporin (**1**).

**Table 4:** Chirality parameter *Q*, calculated for compounds **1** and **2** by averaging over conformers. Conformer geometry and probabilities obtained by DFT calculations were used, unless otherwise specified. In the last column the HTPs measured in the nematic phase E7 are reported.

compound	method	*Q*/10^−3^ nm^3^	HTP/μm^−1^

**1**	B3LYP/6-31g(d,p)	−7	−12
**1**	M06-2X/6-31+g(d,p)	−15	
**1**	NOE^a^ [31]	−19	
**2**	B3LYP/6-31g(d,p)	2	+54
**2**	M06-2X/6-31+g(d,p)	11	
**2**	NOE^a^ [19]	17	

^a^Conformer geometries obtained by DFT/M06-2X/6-31+g(d,p) calculations and distribution derived from NOE experiments.

A possible origin of the lower twisting ability of **1** in comparison to **2**, found in experiments, could be atropisomerization. As a consequence of this process, the sample would contain both cercosporin and its atropisomer. Whereas the former induces a left-handed twist of the nematic director, the latter, having *P* axial chirality, is expected to induce a twist in the opposite sense, with the net effect of lowering the HTP of this dopant. Although atropisomerization is known as a very slow process, our hypothesis is supported by the finding that its rate is significantly higher for cercosporin than for phleichrome [31].

## Conclusion

We have performed HTP measurements, showing that the natural products cercosporin (**1**) and phleichrome (**2**) induce a left-handed and right-handed twist of the nematic director, respectively. This is exactly what is expected for molecules with fused aromatic rings arranged in a helical fashion, having *M* and *P* helicity, respectively. Thus chirality induction in liquid crystals appears to be a suitable technique to determine the axial configuration of perylenequinones.

The integration of experiments with the results of calculations at different levels has allowed us to gain an insight into the conformational preferences of the systems under investigation and into the role of configuration and conformation in determining their twisting ability. We have evaluated the contribution of molecular structures, differing in the conformation of the chiral hydroxyalkyl chains, to the twisting ability of compounds **1** and **2**. Comparing the behavior of these molecules to that of their bare aromatic cores, we have shown that the substituents, although they do not change the sign of the HTP, affect its magnitude.

Our study has evidenced the difficulty of obtaining reliable estimates of the conformational distribution of the perilenequinones by DFT calculations in vacuum and the extreme sensitivity of the results to the choice of the functional and the basis set. In particular, the B3LYP/6-31g(d,p) level was found to be fully inadequate to account for the relative stability of the conformers. Better results were obtained by using M06-2X, a recently developed functional that is more suitable for the treatment of dispersion interactions, and by augmenting the basis set with diffuse functions.

## Experimental

### Helical twisting power measurement

Cholesteric pitch and handedness were obtained at *T* = 298 K by using the lens version of the Grandjean–Cano method [38–39]. The commercially available (Merck) nematic solvent E7 (nematic–isotropic transition temperature *T*_NI_ ~ 330 K) is composed of a eutectic mixture of cyanobiphenyl and terphenyl compounds [30].

#### DFT calculations

Atomic coordinates and energy of the conformers of **1** and **2** were obtained by geometry optimization in vacuum, by using DFT at the B3LYP/6-31g(d,p) [32] and M06-2X/6-31+g(d,p) levels [33]. In each case, the starting geometry was defined by suitably adjusting the conformation of the methoxy groups and of the hydroxyalkyl side chains.

#### SC calculations

The chirality parameter *Q* of a given conformer is defined as


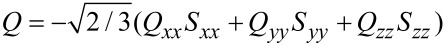


where *S_ii_* is the *i*th cartesian component of the Saupe ordering matrix, which specifies the degree of alignment of the *i*th molecular axis to the local director, and *Q_ii_* quantifies the helicity of the molecular surface, as viewed along the same axis. The Saupe matrix ***S*** and the surface chirality tensor ***Q*** of single conformers were calculated as explained in [40], giving the parameter ξ, which quantifies the orienting strength of the medium, the value 2.5 nm^−2^.

The molecular surface, needed to calculate the ***S*** and ***Q*** tensors, was generated on the basis of atomic coordinates by using the program MSMS [41]. We assumed the following set of van der Waals radii: *r_H_* = 0.1 nm, *r_O_* = 0.15 nm and *r_C_* = 0.185 nm [42], along with a rolling sphere radius equal to 0.3 nm [40] and a density of vertices equal to 500 nm^−2^.

The chirality parameter of a given compound *Q*, was calculated as


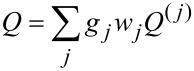


where the sum is over all the conformers and *g_j_*, *w_j_* are the degeneracy and the probability of each of them, respectively. The probability is defined as


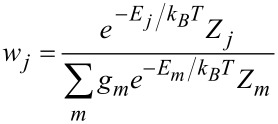


where *k_B_* is the Boltzmann constant, *T* is the temperature, *E_j_* is the potential energy of the *j*th conformer, obtained by DFT calculations in vacuum, and *Z_j_* is its orientational partition function. This accounts for the stabilization of the conformer in the nematic environment and is defined as


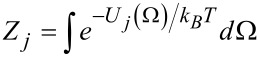


where *U_j_*(Ω) is the orientational potential experienced by a dopant, in the orientation defined by the Euler angles Ω, inside the liquid-crystal phase [29,40].
